# Soccer and Sudden Cardiac Death in Young Competitive Athletes: A Review

**DOI:** 10.1155/2013/967183

**Published:** 2013-03-26

**Authors:** John P. Higgins, Aldo Andino

**Affiliations:** ^1^Exercise Physiology, Memorial Hermann-Texas Medical Institute, The University of Texas Health Science Center at Houston, 6431 Fannin, Houston, TX 77030, USA; ^2^Lyndon B. Johnson General Hospital, The University of Texas Medical School at Houston, UT Annex-Room 104, 5656 Kelley Street Houston, TX 77026, USA; ^3^The University of Texas Medical School at Houston, 6431 Fannin, Houston, TX 77030, USA

## Abstract

Sudden cardiac death (SCD) in young competitive athletes (<35 years old) is a tragic event that has been brought to public attention in the past few decades. The incidence of SCD is reported to be 1-2/100,000 per year, with athletes at a 2.5 times higher risk. Soccer is the most popular sport in the world, played by people of all ages. However, unfortunately it is cardiovascular diseases such as hypertrophic cardiomyopathy and arrhythmogenic right ventricular cardiomyopathy that have subtly missed screening and claimed the lives of soccer stars such as Marc Vivien Foe and Antonio Puerta during live action on the field and on an internationally televised stage. This paper covers the physiological demands of soccer and the relationship between soccer and SCD. It also reviews the most common causes of SCD in young athletes, discusses the current guidelines in place by The Fédération Internationale de Football Association (FIFA) for screening among professional soccer players, and the precautions that have been put in place to prevent SCD on the field in professional soccer.

## 1. Introduction

Due to its tragic and unexpected nature, sudden cardiac death (SCD) in athletes has been brought to public attention in recent decades. The incidence of SCD has been reported to be approximately 1-2 per 100,000 person-years, with a 2.5 times higher risk in athletes when compared with nonathletes [1]. SCD in athletes is most commonly due to congenital and/or acquired cardiovascular disease. Different studies have reported various cardiac diseases as the most common cause. In studies done in the United States, hypertrophic cardiomyopathy (HCM) was the most common cause, followed by congenital coronary artery anomalies (CCA), myocarditis, and arrhythmogenic right ventricular cardiomyopathy (ARVC); ion channelopathies such as long QT and Brugada syndrome were also identified [2]. SCD can also be induced by a traumatic blow to the chest (commotio cordis). Also, the incidence of SCD is known to be greater in males than females [1–4].

Soccer is the most popular sport in the world, played by men, women, and children of all ages [5]. Unfortunately, soccer has been plagued in recent years by deaths at the professional level during internationally televised games. This paper aims to review the physiology of soccer, the relationship between SCD in soccer and other sports, the commonest causes of SCD in young athletes, and the current screening guidelines in place by The Fédération Internationale de Football Association (FIFA) for soccer.

## 2. Methods

Searching primarily the PubMed database, a search of American and International articles available in English was performed. There was no definite time period set due to the limited studies on SCD and sport, but keywords were used to find peer-reviewed articles and case studies on the current reporting of soccer and SCD. Keywords used in the search were *soccer, soccer cardiac, soccer and sudden cardiac death, and athlete sudden cardiac death.* Authors in the selected studies defined SCD as cardiac arrest during or within 1–12 hours of competition [1–4, 6–17]. A young competitive athlete was defined as an individual <35 years old who participated in 2 h a week of physical training or participated in organized team or individual sports that required regular competition against others as a central component, placed a high premium on excellence and achievement, and required systematic and, in most instances, vigorous training [2, 18]. While some studies included subjects over the age of 35, the results focused on subjects from those studies under the age of 35. This paper aimed to highlight the reported causes of SCD in young competitive soccer players, as well as the most common causes of SCD in young competitive athletes overall. 

## 3. Physiology of Soccer

The game of soccer relies on aspects of psychological, physiological, and biomechanical/technical skill. Players at the highest levels of competition master all of these components, but players of all levels excel at certain skills over others. However, in recent years there has been a trend towards selecting players with more favorable anthropometric profiles to compete at the highest level [19].

Studies conducted by filming soccer players found that distances covered at the top level during a 90-minute game are about 10–12 km for players on the field and 4 km for the goalkeeper [19]. During a soccer match, sprinting occurs approximately every 90 seconds and lasts 2–4 seconds [20, 21]. The game is dynamic considering that in addition to running, there is also twisting of the torso to change direction, heading, tackling, and holding the ball against defensive pressure. Energy production while playing soccer is mainly dependent on aerobic metabolism; however, the work intensity can approach the anaerobic threshold, which is defined as the highest exercise intensity where the production and removal of lactate are equal (usually at 80–90% of maximum heart rate) [19].

## 4. Incidence of Sudden Cardiac Death in Soccer Players

Table 1 lists the cardiovascular diseases that have been reported in SCD while playing soccer and other sports. The listed results focused on athletes under the age of 35, since it is known that athletes over 35 experienced SCD most commonly due to atherosclerotic CAD [1, 2, 4]. Several of the studies in Table 1 report soccer as the most common sport associated with SCD [1, 3, 4, 6]. However, it is only the study done by Corrado in the Veneto region of Italy that reported that the incidence of SCD associated with soccer was not statistically significant when compared with other sports, nor was any sport associated with a specific form of fatal disease [1]. In a study done on SCD in Spain, the subjects under 30 were not found to have any predominant cause of SCD associated with soccer [8]. In another worldwide study where soccer was found to be the most common sport associated with SCD, the authors concluded that this was simply due to this sport being played more than others in regions from which the studies were reported and that in fact it was most likely the sports with high cardiovascular demand and isotonic work that put athletes at greatest risk for SCD [4].

## 5. Causes of Sudden Cardiac Death in Soccer Players

### 5.1. Hypertrophic Cardiomyopathy (HCM)

On June 26, 2003, an internationally televised semifinal match was played for the FIFA Confederations Cup between Columbia and Cameroon at Stade de Gerland in Lyon, France [22]. During the 72nd minute of the match, Marc Vivien Foé, a 28 year-old veteran midfielder of the Cameroon national soccer team collapsed on the center circle [23, 24]. Attempts to revive him with CPR were started on the field, and after 45 minutes of resuscitation efforts, he died shortly after arriving to the stadium's medical center. On autopsy, he was found to have HCM [25]. 

HCM is known to be the most common cause of SCD in young athletes in the United States [2]. It has a prevalence of 1 in 500 in several countries including USA, Europe, Japan, China, and East Africa [26]. The pathophysiology lies in autosomal dominant mutations in 11 or more genes encoding thick and thin contractile myofilament protein components of the sarcomere or the adjacent Z-disc [26]. These mutations lead to the histopathological finding of myocyte disarray [26].

Suspicion for this diagnosis is suggested by cardiac symptoms, with the findings of a murmur or abnormal electrocardiogram. Abnormal ECG patterns are present in the majority of HCM patients (75–95%); these findings include markedly increased *R*- or *S*-wave voltages, deep and prolonged *Q*-waves, and deeply inverted *T*-waves [27]. The diagnosis is confirmed by 2D echocardiogram or cardiovascular MRI [26]. Imaging findings show an absolute increase in the left ventricular wall thickness (to 21-22 mm on average), which can also be associated with mild right ventricular hypertrophy [26]. The cause of death in these patients is usually ventricular fibrillation and other tachyarrhythmias [26]. Athletes who are suspected to have low-risk HCM can participate in leisurely sports with yearly followup; qualifying criteria includes no SCD in relatives, no symptoms, no LVH or ventricular arrhythmias, and normal diastolic filling/relaxation [28]. Athletes confirmed to have HCM with any of the aforementioned characteristics are to be restricted from competitive sports as it is believed that high-intensity sports predispose to subendocardial ischemia that leads to ventricular arrhythmias [28].

### 5.2. Congenital Coronary Artery Anomalies

A study by Corrado showed that CCA were associated with the highest risk of SCD in young competitive athletes, and various studies have reported that CCA are the second most common cause of SCD in athletes under 35, associated with 15–25% of cases [1, 2, 4, 29–31]. Table 1 lists case studies of SCD in soccer that have been found to be due to CCA on autopsy. Studies have shown that the most common malformation reported in SCD series both in the young and in the athlete is the origin of a coronary artery from a wrong aortic sinus of Valsalva, either the right from the left coronary sinus or the left from the right coronary sinus, with a proximal course between the aorta and the pulmonary trunk [32]. Patients usually present with cardiac symptoms including exertional syncope and chest pain [32]. The diagnosis is most commonly confirmed by transthoracic echocardiography in children and is supplemented by MRI and CT angiography [33]. Timely diagnosis of CCA is critical because (1) athletes must be restricted from competitive activity to prevent SCD and (2) CCA are surgically correctable [32].

### 5.3. Arrhythmogenic Right Ventricular Cardiomyopathy (ARVC)

On August 25, 2007, a soccer game between La Liga Spanish teams Sevilla and Getafe was played at Sánchez Pizjuán Stadium. During the 35th minute of the game, 22-year-old Sevilla defender Antonio Puerta crouched next to the penalty box, then collapsed [34]. He was found to be in cardiac arrest and was resuscitated on the field. He was substituted and managed to walk off the field, when he reached the locker room he collapsed again, and was pronounced dead at Virgen del Rocío University hospital; his autopsy revealed ARVC [34].

In the Veneto region of Italy, ARVC is the cardiovascular disease that conveys the second highest risk of sports related sudden death, and in Spain it was found to be a predominant pathology associated with SCD in athletes <30 years old [1, 8]. It is currently estimated that disease prevalence is between 1 in 2000 and 1 in 5000 [35]. ARVC is characterized by structural and functional abnormalities of the right ventricle, ranging from regional wall motion abnormalities and ventricular aneurysms to global ventricular dilation and dysfunction; it may also involve the left ventricle [36]. The clinical picture is usually dominated by ventricular arrhythmias that lead to SCD. Symptoms of ARVC include palpitations, syncope, cardiac arrest, or SCD in adolescents or young individuals [36]. The presence of *T*-wave inversions in V1-V3 or premature ventricular complexes (PVCs) of LBBB morphology on 12 lead ECG are the clues noted during cardiovascular screening [36]. However, less commonly patients may present with what appears to be congestive heart failure (CHF) due to DCM [36]. Patients with ARVC should not under any circumstance be allowed to participate in competitive sports in order to prevent SCD [28, 36].

### 5.4. Commotio Cordis

Dundela F.C. is a Northern Irish intermediate-level professional soccer team. On August 25, 1995, the team's captain Michael Goddard was struck on the chest by a ball and collapsed; he was found to be in cardiac arrest and died shortly afterwards [37].

Commotio cordis is defined as when blunt trauma to the chest leads to ventricular fibrillation and therefore cardiac arrest (most commonly during the *T*-wave upstroke on ECG, causing a PVC, which leads to ventricular fibrillation) [38]. Due to a rise in reported incidents, a study recently done by Maron compared the international cases of commotio cordis with those inside the US. The results of this study showed that in both groups commotio cordis occurred among young males and that resuscitation and defibrillation rates did not differ between US and non-US subjects [3]. Although a difference was found in the sports involved: in the US the sports most commonly involved were baseball/softball and American football, in non-US subjects the most common sport was soccer, followed by cricket and hockey [3]. A notable finding in the international subjects was that in seven of the cases involving soccer, a traumatic blow caused by a soccer ball to the chest led to commotio cordis [3]. This finding contradicts the previous notion that air-filled projectiles conveyed a lesser risk than those with a solid core (baseballs, lacrosse balls). However, this study is limited by the fact that the data is predicated on incidents reported to the commotio registry and may not represent all cases of commotio cordis worldwide [3].

### 5.5. Cases without a Known Cause

In the aforementioned studies reviewing SCD and sport, there has always been a percentage of the sample in which the cause of SCD could not be determined by autopsy. However, the reports have been largely variable, ranging anywhere from 1% to 16.3% [1, 2, 4, 7, 8]. A study in the UK reported soccer as the most common sport associated with SCD in ages 11–35, but the autopsies of athletes from all sports had a morphologically normal heart in 23% of cases [6]. Studies done in the US and UK with the families of an individual who experienced SCD found that in 40–50% of families studied, relatives had an ion channel disorder such as catecholaminergic polymorphic ventricular tachycardia, long QT syndrome, and Brugada syndrome [39, 40].

In soccer, like all other sports, doping has become a relevant issue, as evidenced in the 1994 World Cup when the Argentinian superstar Diego Maradona was expelled from the tournament after testing positive for several banned substances [41]. However, the studies that have been mentioned either excluded toxicological confounders as a cause for SCD, or had such a small percentage of cases that they were not addressed [1, 2, 6–8]. Androgen abuse has been shown to have the direct effects of cardiac hypertrophy and myocardial fibrosis, and indirect effects of hypertension, dyslipidemia, arrhythmia, and myocardial infarction [42]. One study reviewing the autopsy findings of four body builders who experienced SCD found cardiac hypertrophy and fibrosis in the myocytes of these subjects [43]. Other medications that may play a role in soccer players' SCD include but are not limited to NSAIDs, antihistamines, and herbal supplements. Of note, COX-2-selective NSAIDs prescribed for musculoskeletal and arthritic complaints have been shown to have an increased risk of adverse cardiac events [44]. Furthermore, some second-generation antihistamines such as terfenadine and astemizole have been shown to reach high serum levels through drug and food interactions thereby predisposing to QT prolongation and ventricular arrhythmias [45].

## 6. Discussion 

The studies reviewed above show that in Italy and much of the rest of the world, the sport in which SCD occurs most often is soccer [1, 6]. However, in the United States, the sports most commonly associated with SCD are basketball and American football [2]. Therefore, it is reasonable to conclude that the regional difference in SCD and sport are likely due to the most common sport being played and that it is the increased cardiovascular demand that predisposes to SCD rather than the sport itself. This study is limited in that most of the studies reviewed do not report whether there is in fact a correlation between soccer and a form of SCD. However, the one study that did assess this variable was the study done in the Veneto region of Italy, which found no statistical significance between the incidence of SCD in soccer when compared with other sports, nor did it find a relationship between soccer and an underlying cause of SCD [1]. Also, while the sample was considerably smaller, the study of SCD in Spain also did not find a predominant cause of SCD associated with soccer [8].

The most common cardiac abnormality implicated in SCD in young athletes <35 years old in the United States is HCM, followed by CCA and ion channelopathies [2], while in Italy the most common causes of SCD in young athletes were CCA, ARVC, and premature coronary artery disease [1]. A study of SCD in Spain showed ARVC to be the most common cause; however, in the UK isolated LVH (confirmed by microscopy to not have myocyte disarray) was most common, followed by ARVC [6, 8]. These results show that there may be a greater prevalence of ARVC in European nations compared to the United States [1]. It is worth mentioning that preparticipation screening with ECGs in Italy is suspected to be the reason why there is a decreased incidence of SCD due to increased identification and management of HCM [1]. Bille et al. reviewed studies around the world and also found CCA and HCM to be the most common causes of SCD but suggested that these pathologies may be the most common because they are the easiest to identify, and that more occult etiologies such as arrhythmias and ARVC may be underrepresented [4]. As noted previously, in each of the studies discussed there was a percentage of the sample in which no cause was found [1, 2, 4, 6–8]. Moreover, given the high prevalence of ion channel disorders found in family members of individuals who experienced SCD, more research is needed on the efficacy of screening the relatives of these individuals and prevention of SCD [39, 40]. While most studies excluded positive toxicological findings, the cardiac effects of steroids and other medications may predispose to SCD [43–45]. Since soccer players stereotypically have a slender build for speed and agility, steroid use may seem counterintuitive, but some players have tested positive [41, 46]. FIFA which is the worldwide governing body of soccer, has taken a staunch stance against doping and in 2001 suspended legendary Dutch players Edgar Davids and Frank de Boer after testing positive for the anabolic steroid nandrolone [46]. 

In evaluating these studies, sampling bias is an issue considering that the research on SCD is based on autopsy results from reported cases, and there is the possibility that cases may go unreported. There is a need for a mandatory reporting database of these incidents in the United States and other countries. Until there is better reporting of these incidents, the true incidence and etiology of SCD in soccer and other sports will remain unknown.

The current guidelines for preparticipation screening have not reached a consensus; in 2004 and 2005 the European Society of Cardiology and International Olympic Committee published notably similar guidelines, which contrasted the American guidelines [4, 28]. The main difference was the addition of a 12-lead ECG to the history and physical examination. This decision had been based on the study done by Corrado that showed a significantly decreased incidence of SCD due to HCM in the Italian population [1]. However, the low specificity of ECG as a screening tool in an athletic population is a major disadvantage for its use [47–49].

 In 2005, FIFA took action to prevent SCD in soccer. Prior to the 2006 World Cup in Germany, the FIFA Medical Assessment and Research Centre developed and implemented a comprehensive precompetition medical assessment tailored specifically to this population [50]. The cardiovascular screening included a personal and family history, physical examination, a 12-lead resting ECG, as well as an exercise ECG and an echocardiogram. The results showed that cardiovascular preparticipation screening in international elite soccer teams seemed appropriate and that while ECG and echocardiography with further standardization could be useful, exercise stress testing remained questionable. Moreover, it was previously believed that preparticipation screening distressed soccer players due to the fear of being removed from competition. However, a study done with Norwegian professional soccer players found that the players felt more confident after screening and would recommend it to other players [51]. 

Future studies on SCD in soccer and other sports involves more detailed reporting of SCD by cause and associated sport. There was a case by Zeller et al. in Table 1 of SCD in a 26-year-old soccer player whose only suggestive finding was marked early repolarization on ECG [11]. Furthermore, a recent study by Lengyel reported a statistically significant QT prolongation at rest in professional soccer players when compared to aged matched controls [52]. Future studies need to be directed towards identifying further cardiac risk factors that may lead to SCD in soccer players. 

After the death of Marc Vivien Foe in 2003, FIFA reacted by making it mandatory to equip all stadiums with automated external defibrillators (AEDs) as well as to have available medical and paramedical personnel who are able to manage emergencies such as cardiac arrest [53]. A remarkable result of these initiatives can be attributed to the case the 24-year-old Fabrice Muamba, who on March 17, 2012, collapsed on the field during an internationally televised game between Bolton and Tottenham of the English Premier League [54]. Resuscitation began on the field and Muamba is reported to have been in cardiac arrest for 78 minutes and to have received a total of 15 defibrillator shocks. While he has now retired from soccer, Muamba has made a full recovery with no neurological deficits, a medical miracle likely related to the onsite AED and well trained personel rapidly responding to this emergency [54].

## 7. Conclusion 

Because soccer is the most commonly played sport worldwide, more of those considered “at risk” experienced an episode of SCD while playing the sport. Several studies reported that soccer was the most common sport associated with SCD, and that the causes of SCD were similar for soccer as among sporting activities in general (HCM, CCA, and ARVC). Therefore, we conclude that preparticipation screening in soccer players should focus some effort on screening for those structural and/or electrical abnormalities associated with these conditions. Given the popularity of the sport worldwide and recent deaths of soccer players on the field, further research on this topic is encouraged.

## Figures and Tables

**Table 1:** Studies reporting SCD in soccer.

Author	Design	Subjects	Most common sport	Cardiovascular disease found
Corrado et al. [1]	Prospective	23/300 athletes, ages 12–35	(1) Soccer (2) Swimming	(1) CAD^a^ (2) ARVC
de Noronha et al. [6]	Prospective	118 cases, mean age 28	(1) Soccer (2) Running	(1) Isolated LVH^b^ (2) Normal heart (3) ARVC
Maron et al. [3]	Retrospective	60 international athletes (ages 19 ± 3) and 213 US Athletes (ages 14 ± 9 years)	Soccer	Commotio Cordis
Maron et al. [2]	Retrospective	1866 athletes (ages 16 ± 4)	(1) Basketball (2) Football (3) Soccer	(1) HCM^b^ (2) CCA
Bille et al. [4]	Retrospective	388 athletes	(1) Soccer (2) Running (3) Basketball	(1) HCM^b^ (2) CCA
Allouche et al. [7]	Retrospective	32 athletes	(1) Running (2) Soccer	(1) HCM^b^ (2) ARVC
Suárez-Mier and Aguilera [8]	Retrospective	61 athletes, mean ages 31.9 ± 14	(1) Cycling (2) Soccer	(1) ARVC^c^ (2) Isolated LVH
McConnell and Collins [9]	Case study	24 y.o. female	Soccer	Hypoplastic left coronary artery
Chen and Sheppard [10]	Case study	22 y.o. male	Soccer	Ebstein anomaly due to hemangioma
Zeller et al. [11]	Case study	26 y.o. male	Soccer	Marked early repolarization on ECG two weeks prior to SCD; autopsy was normal
Pellissier et al. [12]	Case study	15 y.o. male	Soccer	Left coronary artery arising from the right anterior sinus with an oblique course between the aorta and the pulmonary artery trunk
Pacchioni et al. [13]	Case study	18 y.o. male	Soccer	ARVC
Ronneberger et al. [14]	Case study	8 y.o. male	Soccer	Myxomatous mitral valve with lacerations of the posterior cusp and the left vestibular endocardium and left ventricular hypertrophy
Ottaviani et al. [15]	Case study	13 y.o. male	Soccer	Abnormal origin of left coronary artery from the right aortic sinus of Valsalva
Iskandar and Thompson [16]	Case study	14 y.o male	Soccer	Acute angle takeoff of the left main coronary artery and a transverse slit-like opening with a fibrous cushion, which created a kink near its origin
Meel [17]	Case study	22 y.o. male	Soccer	Abnormal origin of the left coronary artery from the right sinus of Valsalva

^a^These results are for the most common causes of SCD for all sports, not just soccer. Also, coronary artery disease (CAD) refers to both congenital and atherosclerotic disease. While soccer was the most common sport associated with SCD this finding was not statistically significant when compared with other sports, nor was any other sport associated with a specific form of fatal cardiovascular disease [1].

^b^These results are for the most common causes of SCD for all sports, not just soccer [2, 4, 6, 7].

^c^These results are based on all sports in a subset of cases under the age of 30. However, the most common cause of death overall for patients of all ages in this study was CAD. In the cases SCD associated with soccer in subjects under 30, there was no predominant cause, but the causes were 3 LVH, 1 Dilated Cardiomyopathy (DCM), 1 CCA, and 3 undetermined [8].
